# Genetics and Molecular Biology of Epstein-Barr Virus-Encoded BART MicroRNA: A Paradigm for Viral Modulation of Host Immune Response Genes and Genome Stability

**DOI:** 10.1155/2017/4758539

**Published:** 2017-04-28

**Authors:** David H. Dreyfus

**Affiliations:** Clinical Faculty Yale School of Medicine and Keren LLC, 488 Norton Parkway, New Haven, CT 06511, USA

## Abstract

Epstein-Barr virus, a ubiquitous human herpesvirus, is associated through epidemiologic evidence with common autoimmune syndromes and cancers. However, specific genetic mechanisms of pathogenesis have been difficult to identify. In this review, the author summarizes evidence that recently discovered noncoding RNAs termed microRNA encoded by Epstein-Barr virus BARF (BamHI A right frame) termed BART (BamHI A right transcripts) are modulators of human immune response genes and genome stability in infected and bystander cells. BART expression is apparently regulated by complex feedback loops with the host immune response regulatory NF-*κ*B transcription factors. EBV-encoded BZLF-1 (ZEBRA) protein could also regulate BART since ZEBRA contains a terminal region similar to ankyrin proteins such as I*κ*B*α* that regulate host NF-*κ*B. BALF-2 (BamHI A left frame transcript), a viral homologue of the immunoglobulin and T cell receptor gene recombinase RAG-1 (recombination-activating gene-1), may also be coregulated with BART since BALF-2 regulatory sequences are located near the BART locus. Viral-encoded microRNA and viral mRNA transferred to bystander cells through vesicles, defective viral particles, or other mechanisms suggest a new paradigm in which bystander or hit-and-run mechanisms enable the virus to transiently or chronically alter human immune response genes as well as the stability of the human genome.

## 1. Introduction: Overview of EBV Pathogenesis in Autoimmune Diseases

**Figure 1:**
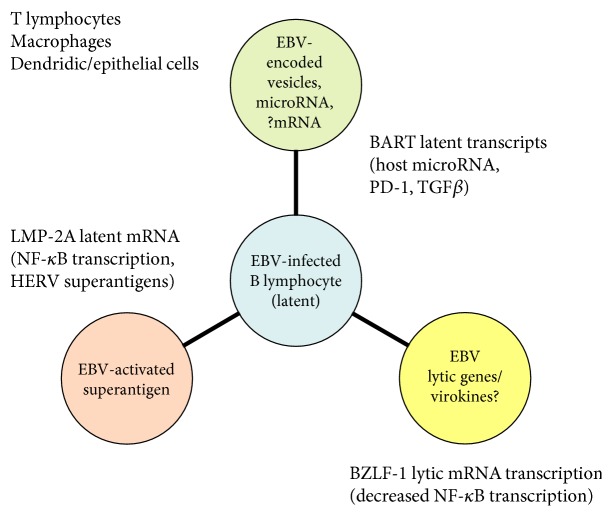
EBV viral genome expression (center) potential for effects on bystander cells of the human immune response are summarized. EBV-encoded BART microRNA may suppress the host immune response and destabilize host genomes in bystander cells through hit-and-run mechanisms during viral latency (top). EBV may also alter the immune response in bystander cells through induced expression of a HERV (human endogenous retrovirus) superantigen by the viral LMP2A protein also expressed during viral latency (left). Induction of viral lytic gene expression by the viral ZEBRA protein may also affect bystander cells through transient expression of viral mRNA as well as viral-encoded micro RNA and viral recombinases and other factors altering host genome stability.

EBV (Epstein-Barr virus or human herpes virus 4), a ubiquitous human herpesvirus, is associated through epidemiologic evidence with common autoimmune syndromes and cancers suggesting that antiviral therapies or vaccines may be a therapy option [1]. Although specific molecular genetic mechanisms of pathogenesis have been difficult to identify, EBV and other common human viral pathogens such as herpes viruses and retroviruses are never cleared from the host and instead enter a latent state which may contribute to viral immunopathology. EBV exhibits complex regulatory interaction with the host genome during latency in part because the virus contains a phenocopy of the host immunoglobulin gene locus and immune response regulatory transcription factors [2–4]. Because of these shared immune response genes, changes in the microbiome, and other environmental factors, the host immune response to EBV and other herpes viruses may be evolving unpredictably with the host immune response (Figure 1).

Although most research on EBV has focused on EBV-infected B lymphocytes which harbor the latent viral genome, in fact, much of the immunopathology related to viral infection may occur due to effects on bystander cells through a variety of mechanisms including viral-encoded microRNA [5, 6]. The author previously suggested a paradigm of “gene sharing” between viral- and host-encoded immune-regulatory genes including viral-encoded cytokines or “virokines,” viral mRNA and microRNA, and human endogenous retroviruses (HERV) which may play a role in immune dysregulation in autoimmune diseases [2]. The author has also previously suggested that EBV may also potentially affect the biology bystander cells such as T lymphocytes through a variety of mechanisms in autoimmune syndromes such as systemic lupus erythematosus (SLE) which are related to EBV infection based on epidemiologic evidence [7]. Since numerous recent publications confirm that viral-encoded microRNA have a complex relationship with host-encoded immunoregulatory microRNA [8–16], and viral-encoded microRNA are themselves apparently coregulated by host and viral immune response transcription factors [17–26], it is reasonable to include viral and host microRNA in the “shared gene paradigm.”

This review is focused upon current peer-reviewed literature regarding the genetics and molecular biology of recently discovered noncoding RNAs encoded by Epstein-Barr virus BARF (BamHI A right frame) termed BART (BamHI A right transcripts) which are suggested as a paradigm for further studies of virus-host interaction. The author will not attempt to review all of the known effects of EBV noncoding RNA, reviewed elsewhere, but instead focus on a specific well-characterized region of the EBV genome, the BamHI A fragment, as a potent source of dysregulation both of host and of bystander cells through as yet poorly understood mechanisms. A review of literature regarding expression of the EBV BamHI A and studies of lytic gene expression of the fragment performed by the author are also included.

A few points regarding EBV genome and nomenclature of the BamHI A fragment may be useful [27]. The virus has a large double-stranded DNA genome of approximately 180 kb with significant differences in substrains due to recombination of internally repeated regions, deletions, and recombinations relative to the canonical strain denoted “B95-8.” Most EBV genome fragments are transcribed bidirectionally arbitrarily denoted “left” and “right.” The large DNA EBV genome was originally digested with BamHI restriction endonuclease to generate a ladder of DNA fragments visible on electrophoresis for mapping studies, with the largest fragment denoted “A” and the smallest “Z,” and this convention has been retained despite more advanced sequencing studies of the viral genome. The author has attempted to include most relevant peer-reviewed published studies available through online search of “PubMed” over the past 2 decades and regrets if inadvertently some references may have been omitted.

## 2. Effects of EBV-Encoded BART MicroRNA on EBV-Infected Cells

The author has chosen to focus upon a particular class of viral-encoded microRNA termed BART because these transcripts are not only expressed in latently infected cells but also found in noninfected cells as will be discussed later in more detail [5, 6]. EBV RNA transcripts from the EBV BamHI fragment termed BART (BamHI right transcripts) and other EBV noncoding RNAs were identified several decades ago and suggested to have regulatory effects on cells prior to the discovery of the host RISC (RNA interference-specific complex). RISC, a member of the DDE magnesium binding nuclease family, was identified as a site-specific nuclease that uses short “hairpin loop” structures in guide sequences termed microRNA to regulate multiple host cell mRNA transcripts. More recent studies confirm that BART form RISC-associated “hairpin loops” that play complex roles in cellular growth and development through inactivation and cleavage of target mRNA by RISC-associated host microRNA homologous sequences in 3′ regulatory regions of the target mRNA [15].

Extensive studies of EBV BART have utilized immunoprecipitation of the RISC and microsequencing of RISC-associated transcripts including BART and host microRNA [15]. These studies have the advantage of providing a complete picture of the viral BART microRNA transcriptome and related host microRNA that may compete for binding sites both at the level of RISC binding and also at the level of binding to host mRNA 3′ regulatory regions. Specific host cell survival factors targeted by BART include the wnt transcription factors, cell cycle growth regulators, and host microRNAs such as miR155. miR155 and related BART have been suggested to be a part of a feedback loop in which most BART are associated with resistance of host cells to apoptosis, but some are opposing and promote apoptosis [28]. Effects of the host transcription factor denoted “NF-*κ*B” which is modulated both by ZEBRA and by other viral latency-associated proteins and may function as a switch coordinated between host and viral genome and between viral lytic and latent growth are summarized (Table 1). NF-*κ*B is also related to expression of human endogenous retroviruses (HERV) and HERV-associated superantigens that in turn may affect cell growth and effects on bystander cells.

As shown in Table 1, significant feedback loops exist in the regulation of BART microRNA since some BART may have effects that oppose cell growth and apoptosis.

Perhaps not surprisingly, the consensus of these studies is that the host BART in general upregulate host pathways providing resistance to apoptosis of the B lymphocyte cell in which the latent viral genome resides [28]. These studies are consistent with the overall effects of EBV latency-associated transcripts and gene products in prolonging the life of the host cell, in contrast to the effects of viral lytic growth factors such as the viral-encoded transcription factor ZEBRA (also termed BZLF-1-encoded protein or ZTA) that promote apoptosis of the host cell to permit viral particle release from apoptotic cells [29–31]. Since the lytic switch protein ZEBRA is coupled to both repression of NF-*κ*B and expression of other transcription sites such as fos/jun, SP1, and CREB, all of these transcription factors in the host response may in turn influence expression of BART.

## 3. EBV-Encoded MicroRNA: A New Paradigm for Viral Immunopathology?

As noted above, significant progress has occurred in the past decade in characterizing effects of EBV on host cell-encoded immunoregulatory microRNA [8–16] and also expression of viral-encoded noncoding and microRNA [17–26]. However, the author and others have previously suggested that much of the immunopathology associated with EBV infection may involve “hit-and-run” effects of EBV gene products transiently expressed in noninfected bystander cells [32]. For example, EBV latent genome products activate expression of endogenous retroviruses or HERV which encode a “superantigen” affecting the host immune response in noninfected lymphocytes and the EBV-encoded ZEBRA protein promotes apoptosis of T lymphocytes required for control of viral replication [1].

Similarly, EBV-encoded BART microRNA as well as other viral-encoded mRNAs could potentially affect bystander cells such as T lymphocytes, macrophages, and epithelial cells through a “hit-and-run” mechanism. BART may be transferred to bystander cells through defective viral particles or vesicles. Bystander cells may have receptors for EBV although these cells are not capable of supporting viral latency. Remarkably, in support of this hypothesis, recent studies of noncoding RNA including microRNA in common human ovarian cancer have recently identified EBV BART in ovarian cancer whole transcriptome sequencing studies [5, 6].

In ovarian cancer, as in other common human cancers, EBV genomes infected cells that are present in malignant tissues but not in all malignant cells. Thus, BART should be included in the effects of EBV on bystander cells affecting the immune response. If in fact EBV-encoded BART can affect expression of other mRNAs in both EBV-infected cells and bystander cells contributing to pathology in common human autoimmune syndromes and cancers, a better understanding of host factors that regulate viral BART may be important, and transcriptional regulation of the EBV BamHI fragment will be the subject of the remainder of this review.

In addition to regulation by NF-*κ*B during viral latency outlined above, the BamHI A fragment encoding BART may also be regulated by viral and host immune response transcription factors during EBV lytic growth (29–31). In contrast to BART which are transcribed in the direction arbitrarily termed “right” during lytic growth expression of the BamHI A fragment is also transcribed to the “left.” Whether left and right transcription of the BamHI A fragment can occur in both directions simultaneously has not been determined since most studies of transcription have focused on latently infected lymphoblastoid cell lines that are stable in in vitro culture rather than in vivo or in primary infection of B lymphocytes in vitro [33, 34]. However, some detailed information is available regarding the transcription of the BALF-2; a left-transcribed open-reading frame adjacent to the BARF is available in peer-reviewed studies [35].

## 4. Regulation of EBV-Encoded MicroRNA BamHI A Fragment by Host and Viral Immune Response Transcription Factors

**Figure 2:**
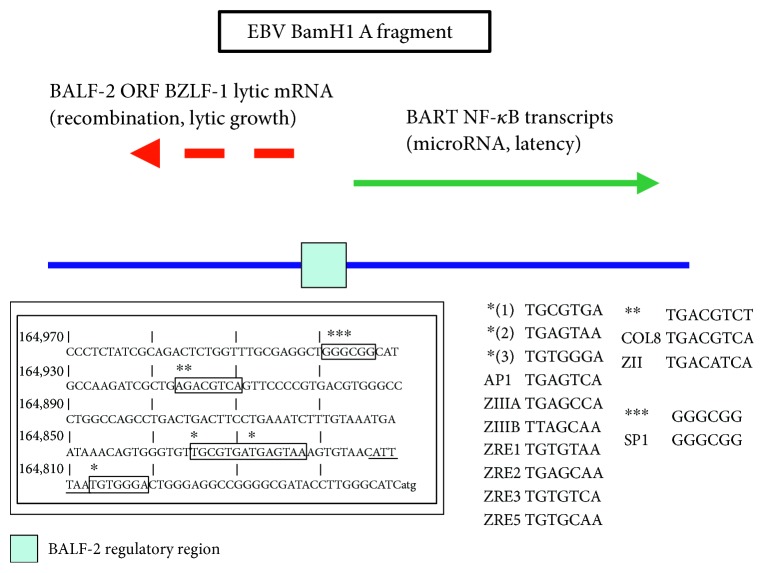
A region within the EBV BART microRNA encoding BamHI A fragment contains regulatory sequences for multiple EBV- and host-encoded immune response transcription factors. EBV genome sequences 164,810 to 164,970 are shown in inverted orientation, with sites for BART also indicated. Sites resembling consensus response elements for CREB, AP-1, and SP1 and a putative TATA box have been identified in the EBV BamHI fragment also encoding BART. AP-1 sites resemble binding sites for host fos/jun factors are similar to sites recognized by the EBV-encoded BZLF-1 (ZEBRA) transcription factor (see text).

Studies of the BALF-2 promoter are important both because of the importance of the BamHI A fragment in regulation of BART and also because the BALF-2 promoter may be coregulated with the human RAG-1 (recombination-activating gene-1) required for generation of the immune repertoire [36, 37]. As shown in Figure 2, a region of the EBV BART microRNA-encoding BamHI A fragment contains regulatory sequences for multiple EBV- and host-encoded immune response transcription factors.

In Figure 2, EBV genome sequences 164,810 to 164,970 are shown in inverted orientation, with sites for BART also indicated. Regions of the EBV BamHI A fragment are located within approximately 10 kb of the BARF genome region of EBV-encoding BART which are transcribed in the opposite direction relative to the BALF-2 mRNA; although because B95-8 has a deletion near the BART locus, exact coordinates between the two strains are not shown. Sites resembling consensus response elements for CREB, AP-1, and SP1 and a putative TATA box have been identified in the EBV BamHI fragment also encoding BART. AP-1 sites resemble binding sites for host fos/jun factors are similar to sites recognized by the EBV-encoded BZLF-1 (ZEBRA) transcription factor.

**Figure 3:**
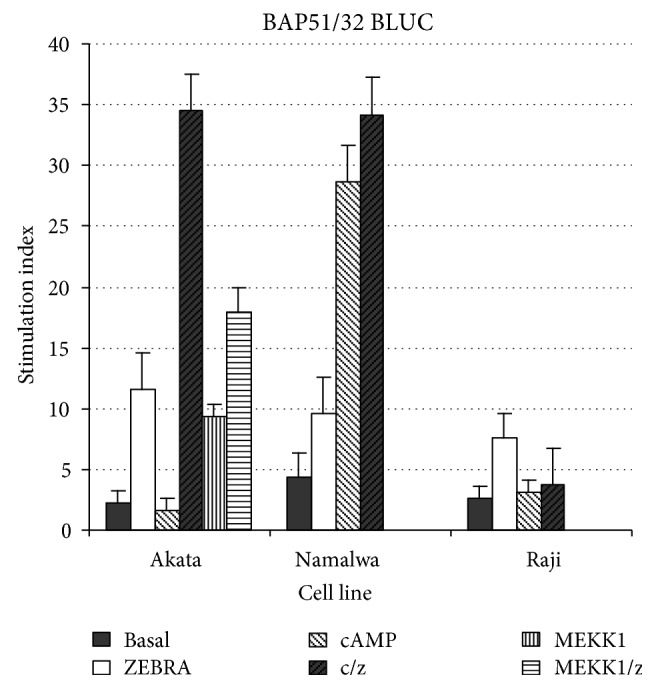
Detection of host immune response transcription factors including AP-1 binding fos/jun induced by cotransfection of cell cycle inducer MEKK1 and viral ZEBRA switch protein are shown in three EBV positive lymphoblastoid cell lines. The promoter region of BALF-2, a 5′ region of the BamHI A fragment adjacent to the BALF-2 mRNA start site, has been cloned from B95-8 viral DNA into a firefly luciferase expression vector (PGL-3 (Promega), reporter vector denoted BAP52/32 BLUC), and sequences have been shown to be identical to those published for B95-8. Results of transient transfection of the putative BALF-2 minimal promoter into 3 EBV positive B lymphoblastoid cell lines (Akata, Namalwa, and Raji) are shown (see text).

In previously unpublished studies by the author, the promoter region of BALF-2, a 5′ region of the BamHI A fragment adjacent to the BALF-2 mRNA start site, has been cloned from B95-8 viral DNA into a firefly luciferase expression vector (PGL-3 (Promega), reporter vector denoted BAP52/32 BLUC), and sequences have been shown to be identical to those published for B95-8. Results of transient transfection of the putative BALF-2 minimal promoter into 3 EBV positive B lymphoblastoid cell lines (Akata, Namalwa, and Raji) are shown in Figure 3. Results shown are a representative of multiple unpublished preliminary results obtained by the author and are similar to published results using a similar region of the BALF-2 promoter in vivo. Results are expressed as the ratio of luciferase activity of BAP52/32 BLUC to control plasmid PGL3-B (denoted stimulation index SI).

As shown, the BALF-2 promoter region of the BamHI A fragment cloned in BAP51/32 BLUC is a functional lytic promoter with SI of 3–5 in unstimulated B lymphoblastoid. BAP51/32 BLUC responds to transient transfection of BZLF-1 with SI of approximately 10 in B lymphoblastoid cells. BAP51/32 BLUC responds variably to activation of the endogenous CREB system (stimulation with dibutyrl cAMP 1 mM). Variable response to CREB activation contrasts with similar responses to BZLF-1 cotransfection in EBV positive B lymphoblastoid cells for unknown reasons. BAP51/32 BLUC also responds to activation of the endogenous AP-1 system (3–5-fold increase in transcription) in B lymphoblastoid cells.

These results demonstrate that the BamHI fragment not only encodes BART microRNA primarily associated with viral latency in one direction (denoted right) but also that transcription of the same region in the opposite direction (denoted left) encodes mRNA for a viral recombinase required for viral lytic replication. An important as yet unanswered question is whether lytic transcripts of the viral BamHI fragment such as BALF-2 and coregulated host RAG-1 transcripts induced by EBV lytic replication are inversely regulated with BART or whether both may be upregulated and expressed in a coordinate fashion by transcription factors such as NF-*κ*B and other host DNA-binding immune response regulatory factors. Another related question is whether BALF-2 mRNA could be coexpressed and packaged into defective viral particles or vesicles and exported to bystander cells contributing to both altered cell growth and genome instability.

## 5. Viral Modulation of Host and Bystander Cell Genome Stability by BART: Implications for Genome Stability

EBV infection is associated with genomic instability of latently infected cells through a variety of previously reported mechanisms [38–41]. Lymphocyte malignancy associated with EBV may also be related in part to expression of EBV noncoding RNA and also an unusual feature of the vertebrate immune system; generation of a large repertoire of T and B lymphocyte receptors through breakage and rejoining of DNA in lymphocytes termed V(D)J recombination [42–45]. While a full discussion of the role of V(D)J recombination and associated RAG (recombination-activating genes) is beyond the scope of this review, EBV infection induces expression of RAG in lymphocytes through unknown pathways [46].

ZEBRA mRNA is detected in cell-free EBV viral particles (unpublished observations by the author) and mRNA for ZEBRA or other viral gene products could be transiently expressed in immune response cells through defective viral particles or other mechanisms. Detection of BART in cells that are not EBV infected also supports the possibility that mechanisms such as defective viral particles and vesicles may actively facilitate transfer of both noncoding and coding RNA to bystander cells.

Remarkably, all herpes viruses share a recombinase termed the herpes DBP (major DNA-binding protein) in the DDE recombinase superfamily with the human RAG-1 recombinase, represented in EBV by the BALF-2 gene product [36]. DBP such as the BALF-2 protein are structurally conserved between widely divergent herpes virus strains and are also absolutely required for lytic replication of known herpes viruses including EBV. These observations support the hypothesis that the DBP shares common regulatory elements (CREB, AP-1, and possibly SP1) with the RAG proteins, providing a plausible mechanism for the activation of RAG-1 transcription associated with EBV infection [46].

The complex relationship between herpes recombinases and their relationship to the RAG-1 gene required for V(D)J recombination and gene sharing between herpes microRNA, recombinases, herpes recombination signals, and V(D)J recombination could provide a link between autoimmune syndromes and malignancy. As discussed in this review, the BALF-2 locus located on the BamHI fragment may also share regulatory signals with the BART locus encoding microRNA that are transported to bystander cells through unknown mechanisms. Thus, it is possible that cotranscription of BALF-2 mRNA with BART microRNA could simultaneously alter the growth and genomic stability of both the host and bystander cells. Since EBV infection of lymphocytes could both alter the immune repertoire of host and bystander cells and also contribute to genome stability of host and bystander cells through expression of both viral induced RAG and viral expressed BART, the author suggests that additional studies of EBV as “hit-and-run” carcinogens are indicated. These studies could also provide new insights into mechanisms of EBV infection as a cofactor in common autoimmune syndromes and suggest new antiviral therapies and vaccine strategies [1].

## 6. Conclusions and Future Research Directions

Gene sharing between human chronic viral pathogens such as EBV microRNA and host cell microRNA may play a prominent role in autoimmune disease and malignancy in the human host (gene sharing between EBV and host proteins; microRNA regulation summarized in Table 2). Simultaneous molecular mimicry response to “altered self” and viral alteration of the host IR (immune response) by EBV could explain why the IR to EBV cross-reactive antigens are not eventually suppressed by the host suppressor cells and cytokines and instead propagate an apparently endless series of autoimmune antibodies and immune dysfunction in various autoimmune syndromes. For example, the author has previously reviewed evidence that common autoimmune syndromes such as SLE (systemic lupus erythematosus), RA (rheumatoid arthritis), and MS (multiple sclerosis) may result from complex interactions between infectious viruses and genes shared with endogenous retroviruses and host genes leading to autoreactive IgG and more recently suggested that chronic idiopathic urticaria, a common cutaneous syndrome with autoimmune features, may also result from similar mechanisms resulting in both autoreactive IgG and possibly other classes of immunoglobulin such as autoreactive IgE [47, 48].

As noted in this review, EBV-encoded BART provide another example of viral genes that are functional homologues of host genes. Unfortunately, gene sharing as a paradigm cannot be studied completely in animal models since EBV and other human herpes viruses do not infect other species, although humanized murine models and other animal models such as rabbits that can become infected with latent EBV may be useful in confirming basic observations such as mechanisms of transfer of BART viral microRNA to host bystander immune cells. Instead, the author suggests that gene sharing between unique human pathogens such as EBV and the human host must be considered as complementary to animal models that consider only host genomic factors rather than viral pathogens and the metagenome. The author suggests that gene sharing between the host IR and viral proteins expressed by latent herpes viruses such as EBV may trigger chaotic behavior in human autoimmune disease through unstable feedback loops and perturbations of immune tolerance associated with human herpes virus reactivation and thus in part explain the failure of animal models to adequately predict response of common autoimmune disease to therapy.

The combined and interactive system of the host genome, the virome, and the microbiome has been collectively termed the “metagenome.” As in astronomy, a system such as the human metagenome with more than two independent variables can become inherently unstable or chaotic. Thus, in actual human autoimmune disease, unpredictable and chaotic interactions between the host genome, the viral genome, or metagenome, and other factors such as the microbiome are evident and must be considered in disease pathology as well as response to disease-modifying agents.

## Figures and Tables

**Table 1:** Summary of EBV gene products related to microRNA gene regulation. EBV gene products modulate levels of host-encoded factors shared with immune response regulatory factors such as NF-*κ*B. Most EBV-encoded BART microRNA are upregulated by NF-*κ*B during viral latency and in turn increase stability of a large number of cellular transcripts that play a role in both resistance of cells to apoptosis and also evasion of the host immune response. EBV-encoded BZLF-1 protein (ZEBRA) downregulates NF-*κ*B and promotes viral lytic growth and host cell apoptosis. Some BART which are homologues of host miR155 may counterregulate other BART and decrease NF-*κ*B. ZEBRA-regulated factors may also upregulate other viral host gene products associated with genome instability (see text).

Transcript	+/−	Effects
BART (1, 2, 3...) microRNA (viral)	NF-*κ*B	Resistance to apoptosis, immune surveillance, host genome instability
LMP2A protein	NF-*κ*B	Resistance to apoptosis, immune surveillance, increased HERV expression
BZLF1 protein (ZEBRA)	NF-*κ*B AP-1 Sp1 CREB	Increased apoptosis, viral replication proteins, viral replication Host genome instability?
BART5-5p miR155 (viral and host)	NF-*κ*B?	Feedback regulation of BART, NF-*κ*B?

**Table 2:** Central role of BART and host transcription factor NF-*κ*B in regulation of host and EBV shared immune response genes (summary).

(i) EBV BARF (BamHI A right frame) encodes multiple viral microRNAs (BamHI A fragment right transcripts) that are important for viral latency and immune evasion.
(ii) Viral LMP-2A, host NF-*κ*B(+) positive regulator of BART, and resistance to apoptosis reversed by viral ZEBRA (BZLF-1) lytic protein.
(iii) BART are transferred to bystander cells by unknown mechanisms, possibly vesicles or defective viral particles.
(iv) Other EBV mRNAs may be transferred with BART microRNA to bystander cells by unknown mechanisms.
(v) Potential for multiple viral regulatory interactions with host and bystanders cell microRNA and gene transcription networks is important in immune surveillance, cell growth, and genome stability.
